# A Novel Approach to Neonatal Resuscitation Education for Senior Emergency Medicine Residents

**DOI:** 10.5811/westjem.2020.10.48623

**Published:** 2020-11-20

**Authors:** Jennie A. Buchanan, Patricia Hagan, Taylor McCormick, Genie Roosevelt, W. Gannon Sungar, Christy Angerhofer, Richard Byyny, Maria Moreira

**Affiliations:** * Denver Health & Hospital Authority, University of Colorado, Department of Emergency Medicine, Denver, Colorado; † Denver Health & Hospital Authority, Department of Pediatrics, Denver, Colorado; ‡ Diversity, Equity, & Inclusion, University of Colorado School of Medicine, Aurora, Colorado

## Abstract

The majority of pediatric visits occur in general emergency departments. Caring for critically ill neonates is a low-frequency but high-stakes event for emergency physicians, which requires specialized knowledge and hands-on training. We describe a novel clinical rotation for emergency medicine (EM) residents that specifically augments skills in neonatal resuscitation through direct participation as a member of the neonatal resuscitation team. The neonatal resuscitation rotation evaluation median score of 4 (interquartile range [IQR] 3,4) was higher compared to all other off-service senior resident rotations combined (median 3, IQR 3,4) for the academic year 2018–2019. Ninety-two percent of residents evaluated the curriculum change as beneficial (median 4, IQR 4,4). The neonatal resuscitation rotation was rated more favorably than the pediatric intensive care rotation (median 4 IQR 3,4 vs median 3, IQR 2, 3) at a tertiary care children’s hospital during the third year. Residency programs may want to consider implementing a directed neonatal resuscitation experience as part of a comprehensive pediatric curriculum for EM residents.

## BACKGROUND

Children account for ~25% of emergency department (ED) visits in the United States.^1^ The vast majority of pediatric visits occur in community EDs, many of which see fewer than 15 children per day in hospitals and lack the resources, personnel, and experience to deliver comprehensive pediatric and neonatal critical care.^1^–^3^ This reality highlights the importance of pediatric readiness for all EDs to stabilize pediatric patients and transfer them to a higher level of care if necessary. In 2009, the major emergency medicine (EM) and pediatric societies jointly published guidelines for pediatric readiness of all EDs, which include proficiency in neonatal resuscitation, stating: “It is essential that hospital ED staff and administrators and EMS systems’ administrators and medical directors seek to meet or exceed these guidelines in efforts to optimize the emergency care of children they serve.”^4^

Caring for critically ill children, and in particular neonates, is a low-frequency and high-stakes scenario for emergency physicians. Pediatric case exposure during residency varies dramatically, and the impact on competence is unknown.^5^ Most EM training focuses on experience in pediatric intensive care units (PICU) and the resuscitations that occur in the ED. However, even EM residents who rotate through high-volume children’s hospital EDs are exposed to very few critically ill patients and get little or no exposure to neonatal resuscitation, a fundamental competency for all emergency physicians to ensure pediatric readiness of all EDs regardless of pediatric volume or acuity.^6^ We describe a novel clinical rotation for advanced EM residents that specifically augments skills in neonatal resuscitation by direct participation as a member of the neonatal resuscitation team.

## OBJECTIVES

Our educational objective was to design a rotation focused specifically on neonatal resuscitations. During this novel rotation, 17 senior EM residents were embedded with the neonatal resuscitation team, attending emergent deliveries and resuscitations.

## CURRICULAR DESIGN

The EM residency is a four-year program with 68 total residents based at an urban, safety-net hospital with adult Level I and pediatric Level II trauma center with a dedicated pediatric ED. The rotation is based at the sponsoring residency institution, an academic safety-net hospital accredited by the Accreditation Council for Graduate Medical Education. The hospital has a Level III nursery staffed by four neonatologists as well as a pediatric intensive care unit (PICU) with 10 beds staffed by four intensivists and an 18-bed inpatient pediatric ward. Senior EM residents receive Neonatal Resuscitation Program (NRP) certification through training specifically tailored for them from the pediatric ED nurse educator and a fellowship-trained pediatric EM attending prior to the rotation. During the weeklong rotation, the resident’s primary responsibility is to participate in neonatal resuscitations with the neonatology team specifically focusing on the critically ill newborn in the first few minutes after birth.

The resuscitation team is composed of the EM resident, neonatal intensive care unit (NICU) advanced practice provider, and nurse (75% of deliveries). A respiratory therapist attends an additional 20% of deliveries if there are prenatal concerns for respiratory distress. For the most complex deliveries (eg, less than 23 weeks gestational age or congenital diaphragmatic hernia), a NICU attending and pharmacist attends. The EM resident is head of bed leading the resuscitation except for the most complex deliveries in which case they would help with assessments. The EM residents attend 3–5 neonatal resuscitations per day, and participate in 3–4 high-fidelity simulation scenarios per day. They also participate in obstetric, PICU and NICU rounds, and may assist with procedures in those units.

Residents are required to give a short presentation on a neonatal resuscitation topic during the week. At the end of the rotation, the residents are expected to set up a neonatal resuscitation, either simulated or in the delivery room, and lead the team through the resuscitation. Previously the senior residents had one week of administration and one week of medical malpractice case review, which were combined into a one-week rotation to allow for the neonatal resuscitation rotation. This research was granted exempt status by the local multiple institutional review boards.

## IMPACT/EFFECTIVENESS

Annually, the EM residents evaluate rotations and changes to the curriculum on a four-point scale (1 = detrimental, 2 = somewhat detrimental, 3 = somewhat beneficial and 4 = beneficial). Evaluation scores are presented as medians with interquartile ranges (IQR). All 17 senior EM residents completed the annual survey. The neonatal resuscitation rotation evaluation median score of 4 (IQR 3,4) was higher compared to all other off-service senior resident rotations combined (median 3, IQR 3, 4) for the academic year 2018–2019. Ninety-two percent of senior residents evaluated the curriculum change as beneficial (median 4, IQR 4,4). The neonatal resuscitation rotation was evaluated as more beneficial (median 4, IQR 3,4) as compared to the PICU rotation (median 3, IQR 2, 3) at a tertiary care children’s hospital during the residents’ third year.

We believe the success of this rotation was due to several factors. The nurse educator for the pediatric ED is a NRP instructor and was willing to provide tailored education to the senior residents along with a pediatric EM attending. The ED and residency program have an excellent relationship with the inpatient pediatric department. The neonatologists and intensivists had previously partnered with us to teach our EM residents during pediatric critical care boot camps. Therefore, the neonatologists had been exposed to the EM residents and had a favorable impression of them prior to the initiation of the rotation.

The neonatologists were very receptive to developing a new rotation specifically addressing the needs of the EM residency program focusing on newborn resuscitation rather than continued care of premature infants in the NICU. Fortunately, there were minimal competing learners for this experience in contrast to many tertiary care NICU and PICU rotations. We believe these factors emphasize the importance of situated cognition in medical education in which learners are more likely to learn when the education provided is within the learners’ practice domain. The needs of the EM residents were recognized as different than the needs of pediatric or neonatology trainees. We have added additional simulation scenarios for the residents to solidify their neonatal resuscitation knowledge throughout their senior year. A limitation of our study is that our primary outcome measure was a subjective satisfaction score.

It is imperative that emergency physicians be competent in pediatric and neonatal resuscitation given that over 90% of pediatric emergency visits will occur in community EDs.^3^ Given the lack of exposure to critically ill pediatric patients in EM training, EM programs need to consider innovative alternatives to provide their residents with a strong foundation for all ages of pediatric resuscitation.
